# From Physiological Spaces to Cognitive Health: A New Perspective on Combined Acupuncture and Chinese Herbal Medicine for Alzheimer’s Disease

**DOI:** 10.34133/research.1467

**Published:** 2026-10-21

**Authors:** Kezhen Zhang

**Affiliations:** ^1^ Institute of Physiological Space Research, Shandong University of Traditional Chinese Medicine, Jinan, Shandong, China.; ^2^ Suzhou Taijitang Traditional Chinese Medicine Hospital, Suzhou, Jiangsu, China.

## Abstract

Alzheimer’s disease (AD) is an escalating global health challenge. Although the target-centric paradigm focusing on targets such as β-amyloid (Aβ) deposition, tau hyperphosphorylation, and neuroinflammation has advanced our understanding of AD, translated therapies remain limited in reversing disease progression. Viewing the brain through the lens of physiological spaces offers an alternative framework for re-evaluating AD pathogenesis. Under this theoretical model, the body comprises both physical structures (“entities”) and fluid-filled interstitial networks (“spaces”), with the latter facilitating metabolic transport, waste clearance, and intercellular signaling. Cerebral perivascular spaces, the extracellular space, and the glymphatic pathway represent specialized cranial interstitial networks. Fluid dynamic impairment within these spatial domains may serve as an upstream trigger for downstream Aβ aggregation, tau pathology, and neuroinflammatory cascades. Combining acupuncture with Chinese herbal medicine may restore fluid homeostasis and spatial patency across these networks. In a preliminary exploration study of 11 patients with mild-to-moderate AD, combined intervention showed encouraging trends toward improving cognitive performance and modulating relevant fluid biomarkers. This spatial perspective provides a complementary target for future AD disease-modifying strategies.

## Limitations of Reductionist and Entity-Centric Approaches

Alzheimer’s disease (AD) is a progressive neurodegenerative disorder clinically characterized primarily by cognitive decline and impaired daily functioning. Globally, approximately 55 million individuals live with dementia, a figure projected to reach 139 million by 2050 [1]. AD pathogenesis involves intricate interactions among β-amyloid (Aβ) deposition, tau hyperphosphorylation, neuroinflammation, and oxidative stress [2]. Conventional pharmacological options, including cholinesterase inhibitors and *N*-methyl-d-aspartate (NMDA) receptor antagonists, temporarily manage symptoms without halting underlying neurodegeneration. In traditional Chinese medicine (TCM), AD falls under the categories of “dementia” and “amnesia”. Pathologically interpreted as kidney essence deficiency combined with phlegm obstruction and blood stasis affecting the cerebral orifices, traditional management focuses on tonifying the kidney, resolving phlegm, removing stasis, and clearing orifices. However, translating these traditional concepts into verifiable mechanistic frameworks remains a challenge.

The slow progress in disease-modifying AD therapies partly reflects the dominant reductionist paradigm in biomedical research. By breaking biological systems down into discrete molecular targets, reductionism assumes that analyzing isolated components reveals full system dynamics. Yet, biological systems exhibit emergent properties that cannot be deduced solely from isolated entities. For instance, higher-order cognitive functions arise from complex network interactions rather than isolated cellular elements. In AD research, target-oriented strategies focusing on Aβ plaques and tau tangles have dominated preclinical and clinical trials [3]. To develop more effective treatments for AD, researchers continue to explore new targets [4]. Despite identifying numerous molecular cascades, targeted therapies have frequently failed to yield proportional clinical benefits. Consequently, exploring broader systemic dimensions, such as fluid dynamics and spatial microenvironments, may offer valuable complementary insights.

## Fundamental Principles of Physiological Spaces

Physiological spaces constitute continuous, fluid-filled interstitial networks distributed throughout the human body. Anatomical entities and their surrounding spaces exist in a dynamic, interdependent state. In the cardiovascular system, for example, vessel walls represent structural entities, while the vascular lumen forms a functional conduit for blood flow. At the microscopic level, interstitial spaces, perivascular spaces (PVSs), and synaptic clefts vary in dimension and composition, yet all function as vital pathways for mass transport, metabolic exchange, and signaling.

Unobstructed interstitial networks sustain tissue homeostasis by enabling continuous nutrient delivery and waste removal. Conversely, structural compression, narrowing, or obstruction of these pathways disrupts fluid transport, laying a common biophysical foundation for diverse systemic pathologies. This concept aligns with the traditional holistic principle that “unobstructed flow maintains health, whereas obstruction induces disease”.

Physiological spaces form a continuous, 3-dimensional interstitial continuum extending from macro-cavities, i.e., thoracic and abdominal cavities down to micro-interstitial and intercellular gap [5]. Recent advances in optical microscopy and in vivo imaging have confirmed the existence of a body-wide, fluid-filled interstitium within human tissues, offering structural evidence for this continuous spatial continuum [6,7]. It should also be recognized that space is not a homogeneous “void” but a dynamic structure filled with interstitial fluid of varying composition and governed by fluid-mechanical principles. Regulation of its volume, pressure, and flow directly influences the microenvironment of the entities it surrounds. This perspective provides another dimension for understanding health and disease: Health depends not only on the normal structure and function of entities but also on the integrity and flow characteristics of physiological spaces. Accordingly, restoration of spatial homeostasis may have broader medical importance [8,9].

In fact, the various intracranial microscopic entities, such as tissues, cells, neurotransmitters, and metabolites, are relatively independent, fragmented entities. They can only be connected into complete, orderly, interactive, and indivisible organic holes via intracranial spaces. Shapson-Coe et al. [10] used electron microscopy to reconstruct the nanoscale ultrastructure of a 1-mm^3^ section of the human temporal cortex, clearly showing extensive microscopic spaces not only between various microscopic entities (e.g., blood vessels, neurons, synapses, and glial cells) but also within them. These spaces enable a clear hierarchical organization among entities and maintain the overall structure in an orderly state, serving as the physical prerequisite for the normal operation of the nervous system.

The spaces between different intracranial cells and tissues are likewise important for maintaining normal nervous-system function. They are also specific manifestations of physiological spaces within the intracranial microenvironment. For example, the neurovascular unit (NVU) is an anatomical and functional unit associated with the cerebral vasculature and consists of neurons, astrocytes, pericytes, endothelial cells, the basement membrane, and microglia, which together coordinate neuronal and vascular functions [11]. The spatial relationships among these cell types integrate cerebral microcirculation with the brain parenchyma into an interconnected and orderly system capable of regulating cerebral blood flow through autoregulation and neurovascular coupling [12]. Meanwhile, the PVSs between cerebral blood vessels and brain parenchyma serve as routes for rapid transport of extracellular fluid within the central nervous system [13]. Normal brain function therefore depends, at least in part, on the integrity of these intracranial microscopic spaces, providing a further perspective for understanding the pathogenesis, diagnosis, and treatment of AD.

## Interstitial Space Dysfunction in AD Pathogenesis

Lacking traditional lymphatic vessels within the parenchyma, the brain relies on specialized pathways to clear metabolic waste. PVSs, the extracellular space (ECS), astrocytic end-feet, and polarized aquaporin-4 (AQP4) water channels collectively constitute the glymphatic system. Cerebrospinal fluid (CSF) enters the parenchyma along periarterial spaces, mixes with interstitial fluid (ISF) within the ECS, and drains along perivenous routes, facilitating the clearance of metabolic products including Aβ and hyperphosphorylated tau. Structural narrowing or functional impairment of these spatial conduits hinders glymphatic clearance, driving neurotoxic protein aggregation, chronic neuroinflammation, and synaptic damage.

As shown in Fig. 1, once physiological spaces are incorporated into the study of AD, the interpretation of the disease, its biomarkers, and its diagnostic and therapeutic paradigms may change accordingly. Within the framework of the theory of physiological spaces, spatial imbalance and structural constraints in the brain microenvironment may represent important initiating factors in a series of pathological cascades. Disorganization of intracranial spaces may impair metabolic waste clearance and microcirculatory perfusion, thereby contributing to chronic neuroinflammation mediated by microglia and astrocytes. This may in turn be associated with Aβ deposition, tau hyperphosphorylation, neuronal structural injury, synaptic dysfunction, and inadequate nutrient supply to brain tissue, ultimately accelerating brain atrophy. From this perspective, restoration of physiological homeostasis in brain spaces may help improve pathways for material transport and metabolism, facilitate the degradation and clearance of neurotoxic proteins and other harmful substances, attenuate persistent neuroinflammatory amplification, and create conditions conducive to structural remodeling and functional recovery of injured neurons. It may also improve microcirculatory perfusion and nutrient delivery in brain tissue, potentially slowing the pathological progression of AD.

**Fig. 1.**
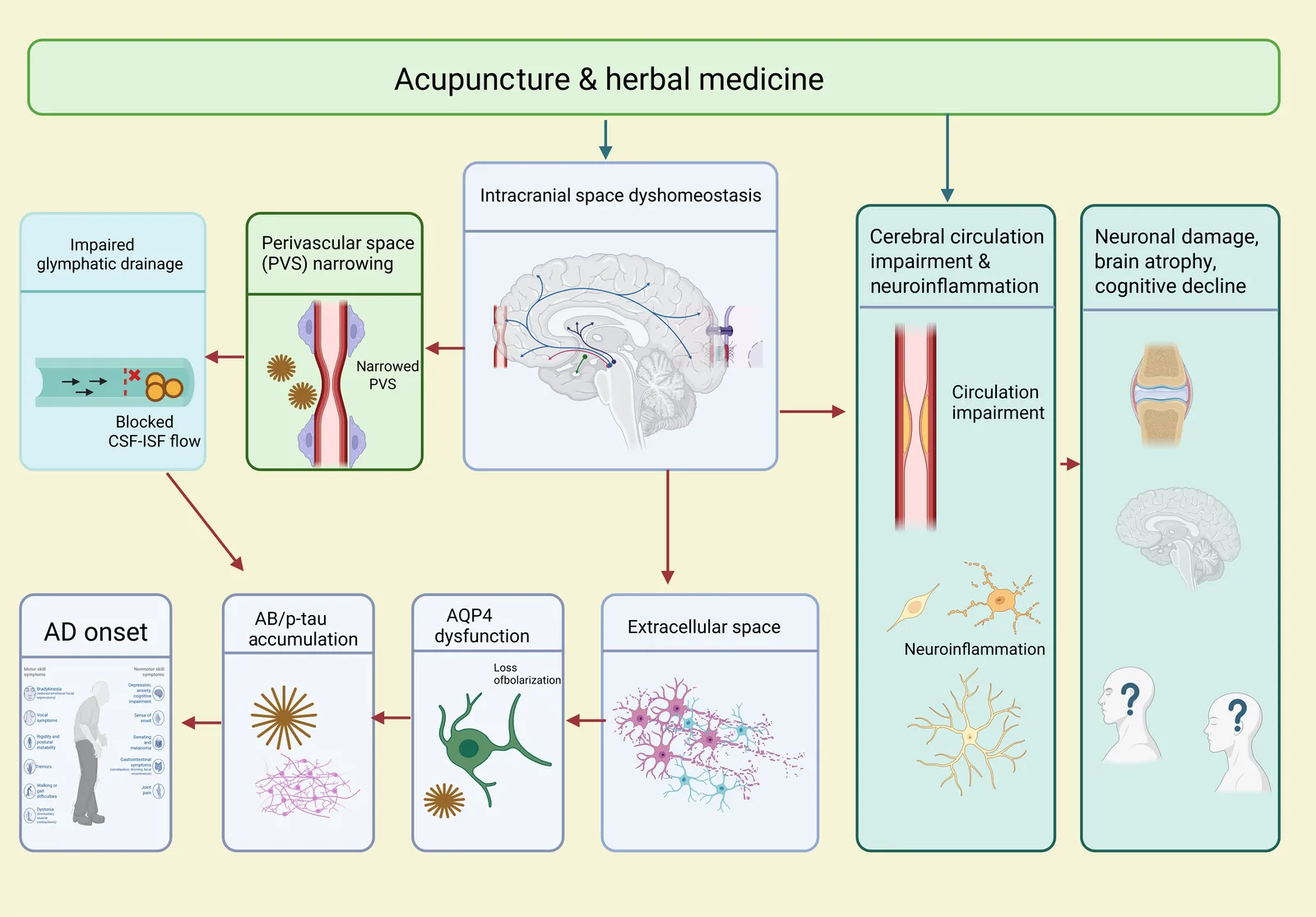
Mechanisms linking brain space dysfunction to the pathogenesis of AD.

The above analysis indicates that although PVS, ECS, and NVU differ in morphology, they share a common feature: They are all interstitial gaps between different tissues and cells within the brain, representing different intracranial manifestations of physiological spaces. Restoring spatial patency may enhance glymphatic drainage, attenuate neuroinflammatory amplification, and improve parenchymal perfusion, creating favorable conditions for neuronal survival and functional recovery.

## Modulation of Physiological Spaces by Combined Acupuncture and Herbal Medicine

Anatomical and physiological investigations indicate that the traditional meridian system closely correlates with interstitial connective tissue planes and fascial spaces [14]. TCM theory holds that meridians “connect internally to the zang-fu organs and externally to the limbs and joints”. This concept describes the meridian system as linking components of the body across macroscopic and microscopic levels into an integrated whole [15]. Meridians can be conceptualized as continuous, low-resistance interstitial conduits that facilitate inter-organ communication and metabolic transport. Anatomical mapping demonstrates that approximately 80% of classic acupoints coincide with intermuscular or intramuscular connective tissue planes [16]. Mechanical stimulation via acupuncture at specific nodes may alter interstitial connective tissue tension, regulate local microcirculation, and influence fluid dynamics across distant interstitial networks, including intracranial spaces [17].

A growing body of research has suggested that meridians possess spatial and channel-like characteristics. The theory of physiological spaces proposes that “meridians are relatively stable and relatively orderly spaces in the human body” [9]. From this perspective, the meridian system resembles a 3-dimensional network of channels linking organs, tissues, and cells into an integrated whole and supporting normal metabolic processes. Within this theoretical framework, narrowing or obstruction of these spaces may be associated with pathological conditions. Research has shown that acupuncture can modulate neurotransmitter levels and improve synaptic plasticity in the brain. Needling selected acupoints along the meridians may also influence and facilitate the flow of physiological spaces throughout the body, including intracranial spaces [18].

Chinese herbal medicine may likewise modulate physiological spaces. Its therapeutic rationale is not limited to intervention at discrete biological targets but is based more broadly on the overall functional state of the individual. Through pattern differentiation, practitioners identify conditions such as cold, heat, deficiency, excess, internal injury, and external pathogen invasion, and select appropriate herbal formulas accordingly. The proposed mechanism is not limited to the action of active constituents on specific receptors or targets of cells or pathogenic microorganisms through biochemical reactions [19]. Rather, treatment considers differences in cold, heat, deficiency, and excess and uses the cold, cool, warm, and hot natures of Chinese herbs accordingly, consistent with the basic therapeutic principles of TCM: “treat cold with heat, deficiency with tonification, excess with purgation, and stasis with unblocking” [20]. In this framework, treatment is tailored to the underlying pathogenesis with the aim of restoring normal physiological function and metabolism. Such treatments often focus on regulating physiological spaces at different levels. For instance, when the body is exposed to cold, it can lead to “wind-cold fettering the exterior”, causing the superficial physiological spaces to contract. The appropriate treatment is to release the exterior with pungent-warm herbs, using Ma Huang and Gui Zhi to restore the superficial physiological spaces. Certain herbs, such as Bo He, Chen Pi, Bing Pian, and Dan Shen, can help unblock obstructed spaces between different tissues and at different levels by regulating and smoothing the flow of qi and promoting blood circulation to resolve stasis. Overall, combined acupuncture and Chinese herbal medicine may restore the flow and functional integrity of intracranial spaces through multiple pathways, potentially enhancing glymphatic clearance, attenuating neuroinflammation, and improving microcirculatory perfusion, thereby creating conditions conducive to structural repair and functional remodeling of injured neurons.

## Preliminary Clinical Observations

To evaluate this spatial paradigm, an exploratory study was conducted involving 11 patients with mild-to-moderate AD who showed inadequate response to standard Western pharmacotherapy (6 males, 5 females; mean age 73.2 ± 5.8 years; disease duration 3.5 ± 1.6 years). While continuing baseline medications, patients underwent a 3-month combined protocol comprising oral modified You Gui Yin decoction (one dose daily), acupuncture [main acupoints: GV20 (Baihui), EX-HN1 (Sishencong), HT7 (Shenmen), ST36 (Zusanli); twice weekly], and topical herbal application. Cognitive outcomes and plasma biomarkers were measured at baseline, 7 d, 14 d, 1 month, and 3 months. All 11 participants completed the study without dropouts.

Following 3 months of treatment, Mini-Mental State Examination (MMSE) and Montreal Cognitive Assessment (MoCA) scores demonstrated progressive improvement from baseline, accompanied by reductions in Clinical Dementia Rating (CDR) and TCM syndrome scores (*P* < 0.001; Tables S1 and S2). Plasma biomarkers revealed a ~21.0% decrease in p-tau181 levels across all 11 subjects and a ~17.7% reduction in glial fibrillary acidic protein (GFAP) in 9 of 11 subjects (Tables S3 and S4). These preliminary findings suggest that targeted spatial interventions may yield systemic benefits across cognitive and biochemical domains.

## Limitations and Future Directions

This preliminary study has several limitations: (a) the small sample size (*n* = 11) and nonrandomized design introduce potential selection bias; (b) the 3-month follow-up period precludes conclusions regarding long-term disease modification; (c) reliance on peripheral blood biomarkers without direct CSF or neuroimaging measures [e.g., diffusion tensor imaging along the perivascular space (DTI-ALPS) for glymphatic flow] limits direct verification of intracranial spatial changes; and (d) the combined treatment design prevents isolating the specific contributions of acupuncture versus herbal therapy.

Nevertheless, as an initial test of this novel hypothesis, this study reexamined the pathogenesis of AD from the perspective of physiological spaces. By employing a combined intervention of acupuncture and Chinese herbal medicine, we observed that patients with mild-to-moderate AD not only showed an improving trend in cognitive function and dementia severity but also exhibited a concurrent decrease in core AD biomarkers in peripheral blood, such as p-tau181. These phenomena suggest that intervening in physiological spaces may exert a holistic regulatory effect on multiple targets, tissues, and systems. Its mechanism of action can be outlined as a progressive process of “unblocking spaces, reconstructing metabolism, and restoring function”, providing a new exploratory angle and broadening complementary strategies for AD treatment.

Ultimately, the scientific and clinical value of the “physiological space hypothesis” depends on its verifiability and falsifiability. Future research can be pursued in the following directions: First, imaging-based evaluation could use magnetic resonance imaging techniques such as DTI-ALPS to quantify glymphatic function and spatial parameters, compare the degree of spatial dysfunction among patients with AD, individuals with mild cognitive impairment, and healthy controls, and examine the temporal relationships among spatial dysfunction, cognitive decline, and changes in Aβ/tau burden. This would help test whether spatial dysfunction precedes the pathological cascade or instead results from pathological changes. Second, animal experiments could manipulate spatial clearance function and determine whether altering PVSs can induce, aggravate, or alleviate AD-related changes, thereby directly testing the causal relationship between spatial dysfunction and Aβ/tau accumulation. Third, intervention studies could evaluate “spatial interventions”, including combined acupuncture and herbal medicine, in randomized controlled trials using factorial designs to distinguish the respective contributions of acupuncture and herbal medicine, while incorporating imaging-derived spatial measures as primary endpoints to test whether restoration of spatial homeostasis can alter disease progression. Fourth, mechanistic studies combining CSF proteomics, multi-omics analyses, and in vivo imaging could clarify the molecular pathways through which spatial interventions may improve the neuronal microenvironment.

If spatial dysfunction is merely an accompanying phenomenon of neurodegenerative changes rather than an upstream driver, then restoring spatial function will fail to alter the disease course, and the hypothesis will be falsified. Conversely, if the hypothesis holds true, spatial function metrics could become complementary tools for the early warning and progression monitoring of AD. Spatially targeted interventions (including but not limited to combined acupuncture and herbal medicine) could synergize with existing anti-Aβ and anti-tau drugs, delaying the disease from dual fronts: “clearing pathological products” and “restoring the spatial background”. The perspective of physiological spaces might also provide a novel analytical framework for other chronic diseases characterized by interstitial network disorders. Regardless of the ultimate outcome, turning our attention to “physiological spaces”, a long-overlooked constitutional element, will help deepen our understanding of neurodegenerative diseases.

In summary, future research should validate the physiological space hypothesis through integrated experimental and clinical approaches. Advanced neuroimaging should assess whether glymphatic dysfunction precedes cognitive decline, while animal models should clarify causal links between perivascular clearance, Aβ/tau accumulation, and synaptic dysfunction. Factorial clinical trials should evaluate the independent and synergistic effects of acupuncture and herbal medicine using glymphatic imaging endpoints, and multi-omics analyses should further elucidate the molecular mechanisms underlying spatial homeostasis and neuroinflammation.

## Ethical Approval

This study was approved by the Ethics Committee of Suzhou Taijitang Traditional Chinese Medicine Hospital (approval no. IRB2026-TCM-01-01) and adhered to the Declaration of Helsinki. Written informed consent was obtained from all participants.

## Data Availability

The data that support this study are available from the corresponding author upon request.
