# Supplementary material for: From Physiological Spaces to Cognitive Health: A New Perspective on Combined Acupuncture and Chinese Herbal Medicine for Alzheimer’s Disease
Source: Research (Wash D C). 2026 Oct 21;9:1467. doi: 10.34133/research.1467 (PMC13683951; doi:10.34133/research.1467)
Supplement: Supplementary file 1 [file research.1467.f1.docx]

Table S1 Results of cognitive function, dementia severity, and Traditional Chinese Medicine syndrome pattern assessments before and after treatment.

| Outcome | Baseline  Mean±SD | Month 3  Mean±SD | Change Mean (95% CI) | % Change | Friedman test | Kendall's W | Adjusted *p* value* | r |
| --- | --- | --- | --- | --- | --- | --- | --- | --- |
| MMSE | 20.36±6.92 | 24.73±5.75 | 4.36  (2.50, 6.00) | 21.4% | *P* < 0.001 | 0.67 | 0.015 | 0.87 |
| MoCA | 15.36±6.01 | 20.73±7.47 | 5.36  (2.00, 8.50) | 34.9% | *P* < 0.001 | 0.78 | 0.014 | 0.88 |
| CDR | 1.73±0.91 | 0.55±0.76 | -1.18  (-1.75, -1.00) | -68.2% | *P* < 0.001 | 0.80 | 0.022 | 0.84 |
| TCM syndrome score | 6.91±4.97 | 1.91±1.70 | -5.00  (-8.50, -3.00) | -72.4% | *P* < 0.001 | 0.79 | 0.023 | 0.83 |

**P* values adjusted using Bonferroni correction for 4 comparisons vs. baseline.

Abbreviations: MMSE, Mini-Mental State Examination; MoCA, Montreal Cognitive Assessment; CDR, Clinical Dementia Rating; TCM, Traditional Chinese Medicine.

Table S2 Analysis of time to onset of effect for each scale.

| Outcome | First significant time point | Adjusted *P* value | r | Median difference |
| --- | --- | --- | --- | --- |
| MMSE | Day 7 | 0.034 | 0.79 | 2.00 |
| MoCA | Month 1 | 0.015 | 0.88 | 4.00 |
| CDR | Day 14 | 0.024 | 0.83 | -1.00 |
| TCM syndrome score | Month 1 | 0.034 | 0.79 | -3.00 |

**P* values adjusted using Bonferroni correction for 4 comparisons vs. baseline.

Abbreviations: MMSE, Mini-Mental State Examination; MoCA, Montreal Cognitive Assessment; CDR, Clinical Dementia Rating; TCM, Traditional Chinese Medicine.

Table S3 Changes in blood biomarkers before and after treatment.

| Biomarker | Baseline Mean±SD | Month 3  Mean±SD | Change Mean  (95% CI) | % Change | Friedman test | Kendall's W | Adjusted *p* value* | r |
| --- | --- | --- | --- | --- | --- | --- | --- | --- |
| p-tau217 | 7.59±3.85 | 6.54±2.72 | -1.05  (-3.07, 0.94) | -13.9% | *P* = 0.237 | 0.13 | >0.999 | 0.34 |
| p-tau181 | 5.29±1.45 | 4.18±1.40 | -1.11  (-1.48, -0.69) | -21.0% | *P* = 0.025 | 0.25 | 0.015 | 0.87 |
| NfL | 65.08±34.14 | 66.19±26.04 | 1.11  (-8.92, 16.15) | 1.7% | *P* = 0.611 | 0.06 | >0.999 | 0.01 |
| GFAP | 230.19±105.57 | 189.39±66.93 | -40.80  (-76.93, -0.24) | -17.7% | *P* = 0.359 | 0.10 | 0.182 | 0.60 |
| Aβ1-42 | 7.18±2.04 | 6.71±1.87 | -0.47  (-1.65, 0.68) | -6.6% | *P* = 0.167 | 0.15 | 0.920 | 0.36 |
| Aβ1-40 | 132.64±19.70 | 142.41±34.13 | 9.77  (-12.33, 45.61) | 7.4% | *P* = 0.027 | 0.25 | >0.999 | 0.04 |
| Aβ42/40 | 0.055±0.014 | 0.048±0.012 | -0.007  (-0.012, -0.002) | -13.0% | *P* = 0.005 | 0.34 | 0.026 | 0.82 |

**P* values adjusted using Bonferroni correction for 4 comparisons vs. baseline.

Abbreviations: GFAP, glial fibrillary acidic protein; NfL, neurofilament light chain.

Table S4 Analysis of time to onset of effect for blood biomarkers.

| Biomarker | First significant time point | Adjusted *P* value | r |
| --- | --- | --- | --- |
| p-tau217 | - | - | - |
| p-tau181 | Month 3 | 0.015 | 0.87 |
| NfL | - | - | - |
| GFAP | - | - | - |
| Aβ1-42 | - | - | - |
| Aβ1-40 | - | - | - |
| Aβ42/40 | Month 3 | 0.026 | 0.82 |

**P* values adjusted using Bonferroni correction for 4 comparisons vs. baseline.

Abbreviations: GFAP, glial fibrillary acidic protein; NfL, neurofilament light chain.
